# Channel surfing: new insights into plasticity of excitation‐contraction coupling

**DOI:** 10.1113/JP277783

**Published:** 2019-03-20

**Authors:** William E. Louch

**Affiliations:** ^1^ Institute for Experimental Medical Research Oslo University Hospital and University of Oslo Oslo Norway; ^2^ KG Jebsen Center for Cardiac Research University of Oslo Norway

**Keywords:** Calcium signalling, Calcium channel, Cardiac myocytes

The heart is a remarkably adaptive organ. This plasticity allows the myocardium to rapidly adjust its function to meet changing functional requirements. For example, during the fight‐or‐flight response, activation of the β‐adrenergic system signals chronotropic, inotropic and lustropic adaptation to meet rising demand. At the level of the individual cardiomyocyte, it has long been understood that this response includes augmentation of Ca^2+^ influx as L‐type Ca^2+^ channels (LTCCs) in the cell membrane are phosphorylated. Ryanodine receptors (RyRs) located adjacent from LTCCs in the sarcoplasmic reticulum are also phosphorylated and sensitized during β‐adrenergic stimulation, and Ca^2+^ stores are increased. Thus, increased Ca^2+^ influx via LTCCs triggers augmented Ca^2+^ release during each action potential, and this larger Ca^2+^ transient boosts contraction of the cardiomyocyte and, ultimately, the whole heart.

In the current issue of *The Journal of Physiology*, Ito and colleagues have provided new insight into how the flight‐or‐flight response is mediated at the level of individual LTCCs (Ito *et al*. 2019). Building on previous findings from their group (Navedo *et al*. 2010), they show that LTCCs are not statically placed in the cell membrane, but rather exhibit dynamic arrangements. They specifically observed that during β‐adrenergic stimulation and phosphorylation by protein kinase A, LTCCs are rapidly mobilized to the membrane from a nearby pool. These newly inserted channels are proposed to join together via their C‐termini, forming functional ‘super‐clusters’ where multiple LTCCs open and close in tandem. The authors linked this so‐called ‘coupled gating’ of neighbouring channels to augmented Ca^2+^ influx; a hallmark of the fight‐or‐flight response.

Several important questions are raised by this work. What is the source of the trafficked LTCCs? Can this pool be enriched or depleted to fine‐tune LTCC availability? And how are the channels in this pool delivered to the membrane? Previous findings have suggested that BIN1 promotes LTCC shuttling to caveolae by tethering microtubules to the membrane. Once LTCCs are delivered, caveolin‐3 is believed to play a critical role in regulating their activity through interactions with protein kinase A (reviewed in Jones *et al*. 2018). It is possible, therefore, that these same mechanisms also promote the rapid delivery, phosphorylation and functional coupling of LTCCs presently described. With a view beyond the fight‐or‐flight response, it seems likely that issues concerning the nanoscale regulation of LTCC position and function have implications for pathophysiology. Indeed, available data have already indicated that there is a caveolin‐3‐dependent loss of L‐type Ca^2+^ current during heart failure, perhaps reflecting a reduction in coupled LTCC gating in this condition. Rather opposite alterations are reported in Timothy syndrome, where an increase in channel clustering and coupled gating has been linked to inappropriate Ca^2+^ entry (Navedo *et al*. 2010). Thus, nanoscale changes in LTCC localization and function may have macroscale consequences, and with better understanding of these mechanisms, it is hoped that LTCC dysfunction can be therapeutically targeted in disease.

The present findings fit well into an emerging understanding of the dynamic nature of excitation‐contraction coupling, and its reliance on channel plasticity. Much of this data concerns the RyR. Indeed, the phenomenon of coupled gating was previously described for RyRs where, interestingly, it was also proposed to occur during β‐adrenergic stimulation (Marx *et al*. 2001). While the mechanisms responsible for coupled gating of RyRs remain unclear, accumulating evidence supports that this gating mode may be specifically linked to the ‘crystalline array’ or ‘checkerboard’ arrangement adopted by neighbouring RyRs after isoprenaline (isoproterenol) treatment (Jones *et al*. 2018). Recent data have indicated that RyRs have surprisingly high mobility within the membrane, and that this ‘channel surfing’ is enhanced when cytosolic Ca^2+^ levels are elevated. This raises the interesting possibility that phosphorylation‐induced changes in RyR open probability might regulate its position, perhaps steering channels into a checkerboard arrangement. As with LTCCs, this malleability of RyR localization has been linked to altered channel activity in disease. Kolstad *et al*. (2018) recently reported that RyRs are dispersed during heart failure, causing channel openings to become uncoupled, resulting in slowed, inefficient Ca^2+^ release during both spontaneous and triggered Ca^2+^ sparks.

Thus far, our understanding of the movement and organisation of LTCCs and RyRs is largely limited to investigations of the two proteins individually. A remaining challenge in the field is to understand how nanoscale plasticity of these channels affects their functional crosstalk. One might hypothesize that β‐adrenergic signalling could enable parallel clustering of the channels on opposite sides of the dyadic cleft, with concurrent coupled gating enabling hyper‐efficient triggering of Ca^2+^‐induced Ca^2+^ release. While speculative, it should be noted that several dyadic regulators, including junctophilin‐2, BIN1 and caveolin‐3 are reported to control the localization of both LTCCs and RyRs. Perhaps these regulatory proteins also fine‐tune their crosstalk? Does loss of these proteins contribute to impaired LTCC‐RyR communication during disease? Addressing these questions will probably require difficult experiments aimed at nanoscale imaging of both proteins simultaneously, perhaps paired with measurements of the ‘gain’ of Ca^2+^‐induced Ca^2+^ release. Nevertheless, the findings of Ito *et al*. (2019) provide an important stepping stone in working towards this important goal.

## Additional information

### Competing interests

None.

### Author contributions

Sole author.
